# Differential Sarcomere and Electrophysiological Maturation of Human iPSC-Derived Cardiac Myocytes in Monolayer vs. Aggregation-Based Differentiation Protocols

**DOI:** 10.3390/ijms18061173

**Published:** 2017-06-01

**Authors:** Dorota Jeziorowska, Vincent Fontaine, Charlène Jouve, Eric Villard, Sébastien Dussaud, David Akbar, Valérie Letang, Pauline Cervello, Jean-Michiel Itier, Marie-Pierre Pruniaux, Jean-Sébastien Hulot

**Affiliations:** 1 Sorbonne Universités, UPMC Univ Paris 06, AP-HP, INSERM, Pitié-Salpêtrière Hospital, F-75013 Paris, France; d.jeziorowska@ican-institute.org (D.J.); v.fontaine@ican-institute.org (V.F.); c.jouve@ican-institute.org (C.J.); eric.villard@upmc.fr (E.V.); sebastien.dussaud@upmc.fr (S.D.); 2 Institute of Cardiometabolism and Nutrition (ICAN), F-75013 Paris, France; 3 Institut du Cerveau et de la Moelle épinière, ICM, CNRS UMR 7225, Inserm U 1127, UPMC-P6 UMR S 1127, Plateforme d’exploration cellulaire, CELIS-Culture Cellulaire, F-75013 Paris, France; d.akbar-ihu@icm-institute.org; 4 Sanofi Recherche et Développement, F-91380 Chilly-Mazarin, France; valerie.letang@sanofi.com (V.L.); pauline.cervello@sanofi.com (P.C.); marie-pierre.pruniaux@sanofi.com (M.-P.P.); 5 Sanofi Recherche et Développement, F-94403 Vitry, France; jean-michel.itier@sanofi.com

**Keywords:** induced pluripotent stem cells, differentiation, cardiomyocytes, sarcomere, cardiomyopathies

## Abstract

Human induced pluripotent stem cells (iPSCs) represent a powerful human model to study cardiac disease in vitro, notably channelopathies and sarcomeric cardiomyopathies. Different protocols for cardiac differentiation of iPSCs have been proposed either based on embroid body formation (3D) or, more recently, on monolayer culture (2D). We performed a direct comparison of the characteristics of the derived cardiomyocytes (iPSC-CMs) on day 27 ± 2 of differentiation between 3D and 2D differentiation protocols with two different Wnt-inhibitors were compared: IWR1 (inhibitor of Wnt response) or IWP2 (inhibitor of Wnt production). We firstly found that the level of Troponin T (*TNNT2*) expression measured by FACS was significantly higher for both 2D protocols as compared to the 3D protocol. In the three methods, iPSC-CM show sarcomeric structures. However, iPSC-CM generated in 2D protocols constantly displayed larger sarcomere lengths as compared to the 3D protocol. In addition, mRNA and protein analyses reveal higher cTNi to ssTNi ratios in the 2D protocol using IWP2 as compared to both other protocols, indicating a higher sarcomeric maturation. Differentiation of cardiac myocytes with 2D monolayer-based protocols and the use of IWP2 allows the production of higher yield of cardiac myocytes that have more suitable characteristics to study sarcomeric cardiomyopathies.

## 1. Introduction

The ability to generate human induced pluripotent stem cells (hiPSC) from patients and the capacity to differentiate these iPSCs into disease-relevant cell types represent a breakthrough for human diseases modeling, preclinical evaluations and drug discovery. In the field of cardiology, the hiPSC technology has been successfully used to model a number of inherited heart diseases, including monogenic channelopathies (notably long QT syndrome [1,2], drug-induced long QT [3] and catecholaminergic polymorphic tachycardia [4]), cardiomyopathies due to mutations in mitochondrial (Friedreich’s ataxia [5]) or desmosomal proteins (ARVC [6,7]) and other rare genetic disorders (LEOPARD [8], pompe disease [9], laminopathie s [10]). On the other hand, hiPSC models of sarcomeric cardiomyopathies have been less reported so far [11,12,13,14,15,16]. Sarcomere mutations are associated with hypertrophic and dilated cardiomyopathies, two prevalent inherited cardiac disorders, and constitute one of the most common causes of cardiac sudden death and represent a major cause for cardiac transplantation.

It has indeed been generally considered that directing hiPSC-derived cardiomyocytes (hiPSC-CM) to acquire the requisite sub-cellular and cellular adult myocyte morphology as well as the appropriate protein content and organization was a major obstacle [17]. All current protocols for cardiomyocyte differentiation are based on recapitulating development signals in the embryo that first direct mesodermal fate, induce cardiogenic mesoderm and eventually result in the formation of bona fide cardiomyocytes. Initial protocols have been developed as aggregation-based methods in which undifferentiated cells are forced together as clumps (or embryoid bodies) [18,19]. More recently, monolayer-based methods, in which cells are seeded and differentiated on a culture substrate, have been developed as they achieve higher efficiency in terms of quantity of generated cardiomyocytes [20,21]. Different mesoderm differentiation-inducing molecules have been proposed. In the first days of differentiation, the use of bone morphogenetic protein (BMP) and Activin A has been progressively replaced by the use of GSK-3 inhibitor, such as CHIR, to initially activate β-Catenin nuclear signaling [22]. In addition, different studies have shown the critical role of subsequent Wnt signaling inhibition, which can be achieved with different molecules that can disrupt the pathway at different levels [23]. Notably, some small molecules (such as IWR1) specifically block the canonical Wnt β-catenin signaling pathway while others (such as IWP2) block the Wnt-mediated mechanisms irrespective of pathway mechanisms.

Consequently, different architectural (i.e., aggregation vs. monolayer) and pharmacological strategies can be adopted to generate hiPSC-CM. However, so far, there has been no direct comparison of methods for cardiomyocyte differentiation using the same lines in parallel experiments and assessing their quantitative efficiency but also the qualitative characteristics of generated hiPSC-CM. Here, we directly compared three different popular protocols to generate hiPSC-CM, the classical aggregation-based methods and two monolayer-based approaches differing by the Wnt inhibitor used. In order to limit selection biases in the evaluation, we set up a high-content cell imaging analysis system to systematically evaluate main cardiomyocytes characteristics. Our analyses demonstrate the strong influence of the differentiation method on the sarcomere formation and its maturation.

## 2. Results

### 2.1. Yield of Cardiomyocytes Differentiation Is Higher with Monolayer-Based Protocols

**Figure 1:**
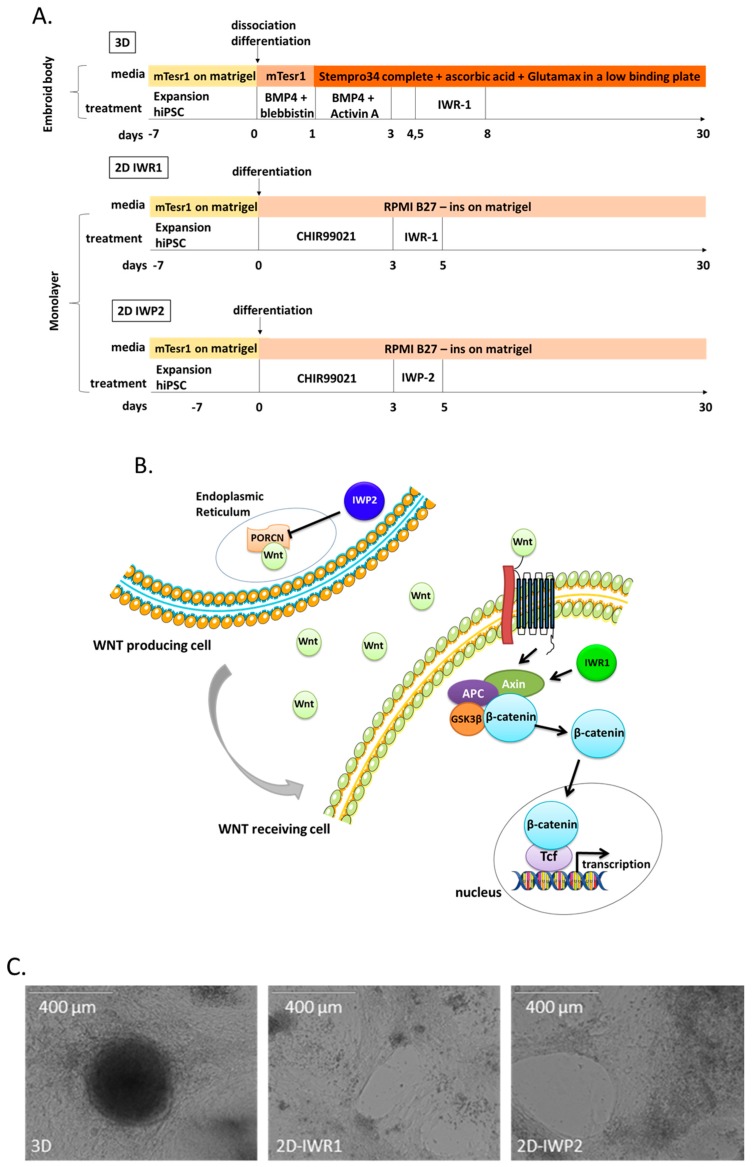

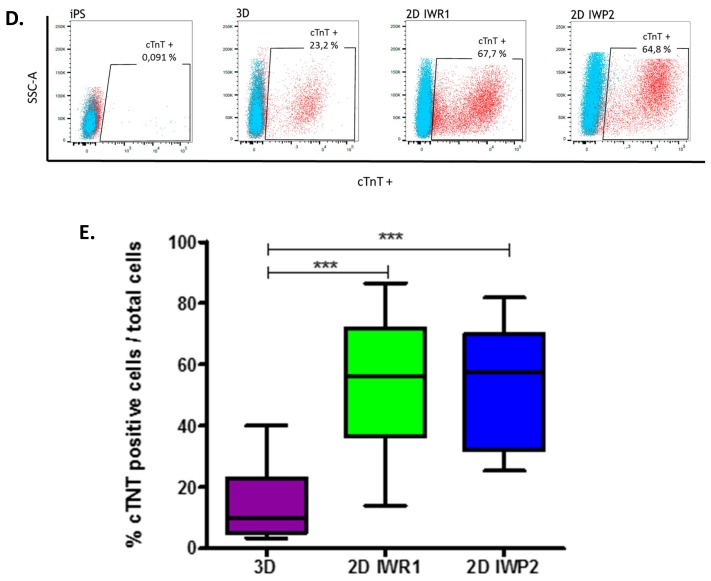
Comparison of protocols. (**A**) Schematic representation of differentiation protocols; blebbistin = blebbistatin (**B**) Schematic overview of the canonical Wnt pathway and the targets of the Wnt inhibitors used; (**C**) Aspect of differentiated iPSCs on day 30 in transmitted light; (**D**) Troponin expression at day 27 ± 2 analyzed by flow cytometry. Typical flow cytometry plots show negative IgG control in blue and cTnT+ cells in red (**E**) Quantification (box-whisker plots showing minimum, first quartile, median, third quartile and maximum) of cardiac troponin T expression measured by flow cytometry in the three compared protocols from 11 to 16 differentiations with 3 different clones. *** *p* < 0.001.

We investigated three differentiation protocols that differed in terms of architectural and pharmacological environment (Figure 1A) in a total of four different iPSC clones (Figure S1). We firstly used a classical aggregation method of EB-formation (3D) where cardiomyocyte differentiation was initiated by enzymatic dissociation and formation of “cardiogenic” embryoid bodies (EBs). The mesoderm progenitors were induced by using a combination of BMP4 and Activin A. Blebbistatin was used to enhance the cell viability (day 0–4.5). Then, cardiogenesis was promoted by inhibiting the Wnt canonical pathway using a small molecule (IWR1), which stabilizes the axin destruction complex (day 4.5–8) [19]. We also tested two different protocols where iPSCs were firstly seeded as monolayer (2D) on matrigel 7 days before mesoderm induction. The induction was obtained by inhibiting the GSK pathway using a small molecule (CHIR99021). Manipulation of the Wnt pathway to promote cardiogenesis was then achieved using two different pharmacological inhibitors: IWR1 (adapted from Burridge et al. [20]) or IWP2 protocol (adapted from Lian et al. [21]) to inhibit Wnt production by inactivating Porcupine, a *O*-acyltransferase responsible for activating Wnt proteins (Figure 1B).

We initially assessed the yield of cardiomyocyte generation using three different iPS clones (31.3, 3.7 and 3.8) that were differentiated at least 10 times overall. We firstly observed that differentiated cells started to beat at day 8 in all protocols (Figure 1C). However, analysis by flow cytometry of cTnT expression at 27 ± 2 days (Figure 1D,E) revealed that the yield of differentiation was similar for the 3 iPSC clones (not shown) but significantly higher with both 2D protocols compared to the 3D protocol (14 ± 11% vs. 54 ± 22% vs. 54 ± 20% for 3D, 2D-IWR1 and 2D-IWP2, *p* < 0.0001).

### 2.2. High-Content Cell Imaging of hiPSC-CM

**Figure 2:**
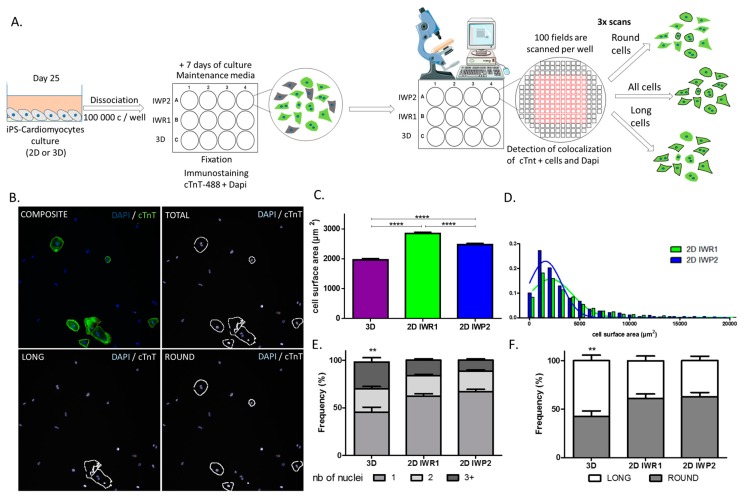
High content cell imaging to analyze the cell size, nucleation and morphology using the cTnT and Dapi staining. (**A**) Design of the automated cellular imaging high content analysis; (**B**) Composite image of the detection of cTnT+ and Dapi+ cells. Detection setting for total, round and long cells (**C**) Cell surface comparison at day 27 ± 2. N > 1500 cells from at least three different differentiations with three different iPSC clones, **** *p* < 0.0001; (**D**) Distribution of cell surface area in generated cTNT+ cells from the 2D-IWR1 (green) and 2D-IWP2 (blue); (**E**) Frequency of number of nuclei in the total cells for each protocol (**F**) Distribution of round and long cells obtained with each protocol. ** *p* < 0.01.

We then designed a high-content cell imaging analysis (Incellsight CX5 system, ThermoFisher Scientific, Waltham, MA USA) that performed a systematic screen of all cells in each culture well. All hiPSC-CM were recognized as co-stained with cTNT and DAPI. This approach was primarily set to systematically quantify cell surface and measure nucleation per cell. We then developed a specific recognition tool to systematically determine the morphology of the analyzed cells (Figure 2A,B).

We firstly observed significant differences in cell surface of generated hiPSC-CM according to the methods. The analysis showed that the cells derived using the 2D protocols were significantly bigger than the cells derived with the 3D protocol (1967 ± 41 µm^2^ vs. 2850 ± 43 µm^2^ vs. 2478 ± 41 µm^2^ for 3D, 2D-IWR1 and 2D-IWP2, *p* < 0.0001, Figure 2C). In addition, hiPSC-CM generated using the 2D-IWR1 protocol were also bigger with a higher dispersion of cell size as compared to the 2D-IWP2 protocol (Figure 2D). We were also able to quantify the number of nucleus per cell in the three protocols. We classified the cells as three groups: mononucleated cells, binucleated cells and cells with 3 or more nuclei (multinucleated). The percentage of each type of cells was calculated. The 2D protocols led to up to 20% of binucleated cells and about 60–65% of mononucleated cells. On the other hand, the aggregation-based 3D protocol was associated with a higher proportion of multinucleated cells (Figure 2E). Finally, the morphology of the generated cells was systematically analyzed by comparison with pre-established cell masks and aspect ratios. Figure 2B shows a typical example of the determination of cell morphology within a given field. We thereby defined two populations: round cells and long cells. We found that the 2D protocols were comparable between each other with 60% of round cells and 40% of long cells (Figure 2E). The 3D protocol however generated 40% of round cells and 60% of long cells. The round and long cells were analyzed for their size and their multinucleation (Figures S2 and S3) showing consistent results with the one observed on total cells. These high-throughput results show that the hiPSC-CM structure significantly depends on the differentiation protocol and suggest a higher inter-cell homogeneity in hiPSC-CM generated using the 2D-IWP2 protocol.

### 2.3. Measurement of the Sarcomere Length

**Figure 3:**
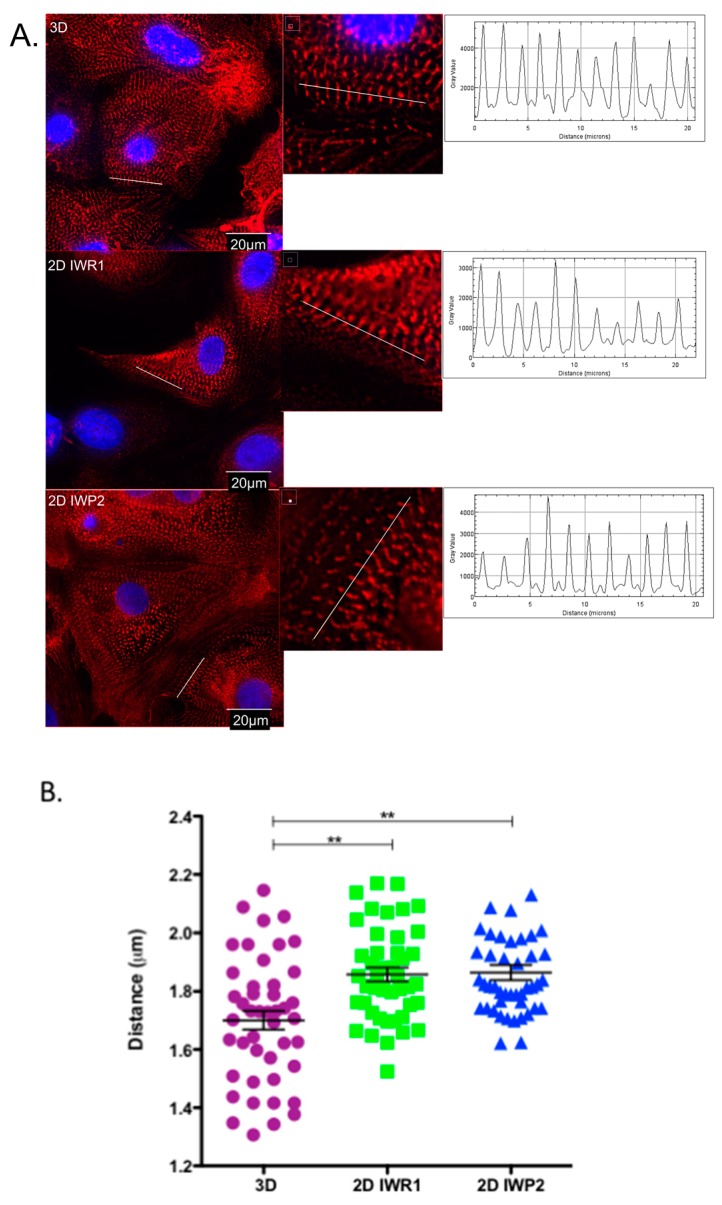
Measurement of the sarcomere length. (**A**) Deconvolution microscopy imaging of hiPSC-CM in the three differentiation protocols. α-sarcomeric actinin is stained in red and nucleus in blue. The sarcomere was measured by tracing a line of 20 µm across the sarcomeres using the Fiji software. The intensity of fluorescence across the line was translated into longitudinal plots; (**B**) Distribution of the sarcomere sizes. *n* = 45 cells for each protocol, from three different differentiations and three different iPSC clones. ** *p* < 0.01.

As anticipated, we found that hiPSC-CM generated with the three protocols displayed typical sarcomeric morphology (Figure 3A). We then measured the respective sarcomere length in hiPSC-CM stained with α-sarcomeric actinin. Measurements were performed in a total of 45 cells (from three different differentiations and three different iPSC clones) for each studied protocol. A 20 µm line was traced across the sarcomeres on myofibrils (Figure 3A). The sarcomere length of the cells generated with both 2D protocols were very similar, ranging from 1.6 up to 2.2 µm with an average size of 1.9 ± 0.03 µm which was close to the sarcomere length of adult cardiomyocytes [24]. The sarcomere length of the cells derived by the 3D protocol was highly variable ranging from 1.3 to 2.2 µm and with a smaller average size 1.7 ± 0.03 µm compared to the one observed with 2D protocols (Figure 3B). The sarcomere length was variable between generated cells but similar for the studied iPSC clones.

### 2.4. Quantitation of Sarcomere Proteins

**Figure 4:**
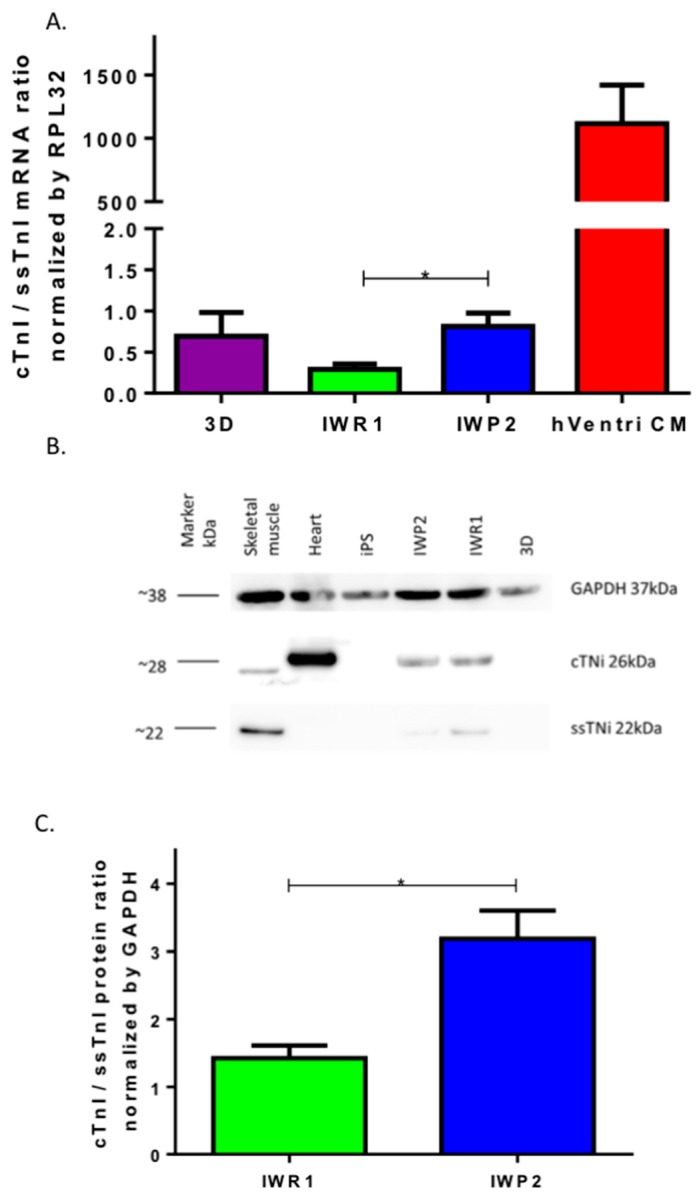
Quantification of sarcomere mRNA and proteins. (**A**) The mRNA expression of cTni and ssTni was quantified by quantitative RT PCR. (*n* = 8 to 12 from four iPSC clones, *p* < 0.05 Kruskal-Wallis and Dunn’s Multiple Comparison Test, hVentriCM was excluded from statistical analysis); (**B**) Typical western blot of cTNi and ssTNi proteins; (**C**) Quantification of the ratio of cTNi/GAPDH on ssTNi/GAPDH proteins in hiPSC-CM generated with the 2D-IWR1 vs. 2D-IWP2 protocols. Quantifications from 4 differentiations with 4 different iPS clones, * *p* < 0.05, Mann–Whitney test.

We then measured the mRNA and protein expression of the different troponins (Tni) isoforms as markers of sarcomere maturation. Cardiac Tni (cTni/*TNNI3*) is a key myofilament protein and is expressed only in adult cardiac muscle. The slow skeletal troponin I (ssTni/*TNNI1*) is another Tni isoform that is expressed in slow skeletal muscle fiber but also in the fetal cardiac muscle. The transition from ssTni to cTni has thus been proposed as a key adult maturation marker [25]. We firstly measured the expression of mRNA of both Tni isoforms and found that cTni:ssTni mRNA ratio was the highest in the cells derived with the 2D protocol using IWP2 as compared to the 2D-IWR1 and 3D protocols (Figure 4A). The cTni:ssTni mRNA ratio was however much lower than the one observed from RNA isolated from human LV tissue as a positive control. Both proteins were then quantified by Western Blot. We used human skeletal muscle tissue as a control for the presence of ssTni and human LV tissue for the presence of cTnI. cTni was detected in hiPSC-CM generated with both 2D protocols but higher ssTni expression was seen in cells generated with the 2D-IWR1 protocol (Figure 4B). Consequently, the cTni:ssTni protein ratio was significantly higher in hiPSC-CM derived from 2D-IWP2 as compared to 2D-IWR1 protocol (Figure 4C).

Strikingly, cTni and ssTni proteins were not detected in hiPSC-CM derived using the 3D protocol. Similarly, the gene expression profiling of key calcium cycling proteins (Serca2a, PLN, CASQ, RYR2) showed a significantly lower expression in 3D-derived cardiomyocytes as compared to both 2D protocols (Figure S4). Altogether, this suggests that monolayer-based differentiation protocols improve sarcomere formation but that the use of IWP2 as a differentiation molecule might be more efficient for sarcomere maturation.

### 2.5. Electrophysiological Characteristics

As we found significant differences in sarcomere formation and maturation, we asked whether differentiation protocols also influenced the electrophysiogical characteristics of generated cardiomyocytes. We firstly analyzed gene expression of main cardiac ion channels (Figure S5A). As compared to 3D protocol, we found that *SCN5A*, *CACNA1*, *KCNQ1* and *HCN4* were more expressed in cardiomyocytes generated with the monolayer-based protocols while *KCNH2* and *HCN2* expression were similar. Of note, expression levels of cardiac ion channels were of the same magnitude than the one observed in human LV cardiomyocytes, while the expression of sarcomeric and calcium cycling genes were globally much lower.

Finally, action potentials were recorded in spontaneously beating cardiomyocytes generated from the three protocols using two different clones (3.7 and 3.8). Recorded parameters are reported in Table 1. All three protocols generate ventricular-like cardiomyocytes as indicated by a ratio APD90/APD50 < 1.3 and APA > 95 mV. The resting membrane potential (RMP) was significantly higher in hiPSC-CM derived with the 3D protocol as compared to the hiPSC-CM obtained with the 2D-IWR1 protocol but not significantly different than the one observed in hiPSC-CM obtained with the 2D-IWP2 protocol. Cardiomyocytes derived from the 2D-IWP2 protocol also presented with a significantly higher APD90 (ms) as well as sodium current (INa, PA/pF).

## 3. Discussion

The cardiomyocytes derived from human iPSC represent a novel cellular platform for modelling cardiovascular diseases. Since the discovery of iPSC in 2006 [26] many methods to differentiate these cells into cardiomyocytes were established and progressively improved. Multiple research laboratories and projects have adopted the iPSC technology and for pragmatic reasons usually select one differentiation protocol to generate cardiomyocytes. However, a parallel and direct comparison of the achievements between protocols has not been performed so far.

In this study, we have compared three protocols [19,20,21] that represent the most frequent methods currently used to generate iPSC-CM. These protocols differ by multiples ways in term of architectural and pharmacological environment. In the 3D protocol [19] the induction into mesoderm is obtained by the activation of the BMP and Nodal pathway which activates the transcription factors of cardiac progenitor cells like GATA4 and NKX2.5. This protocol originates from the seminal report from Keller’s group [18] and has been largely used in different research projects to model cardiomyopathies. Our results suggest that this protocol provides efficient differentiation at the electrophysiological level but not at the sarcomere level, even if these results could be influenced by using an additional purification method to obtain cardiomyocyte enrichment before analysis. In this study, the cardiac progenitors were induced by inhibiting the Axin complex with IWR1, which differs from the original protocol but which has been proposed as a way to obtain more reproducible differentiations [19]. In the 2D protocols, the mesoderm was induced by inhibiting the GSK pathway. The biphasic role of the Wnt/β catenin pathway is known in cardiogenesis [27]. Wnt/β-catenin signaling promotes cardiac differentiation at early developmental stages and inhibits it later. Adding an exogenous Wnt3a in the early stage of differentiation enhances cardiogenesis [28]. The molecule CHIR99021 is a selective inhibitor of GSK3. It promotes self-renewal by stabilizing β-catenin and activating the Wnt/β-catenin signaling [22]. The activation of the Wnt/β-catenin signaling then promotes the activation of early cardiac transcription factors.

We then tested two different pharmacological ways to induce cardiac progenitor cells using monolayer-based approaches as more recently proposed [20,21]. We also compared the characteristics of hiPSC-CM obtained with the use of two different Wnt inhibitors. In the IWR1 protocol [20], we specifically inhibited the canonical Wnt/β-catenin signaling pathway by using a molecule that stabilizes the Axin destruction complex thus leading to β-catenin degradation. On the other hand in the IWP2 protocol [21], the inhibition of Wnt is obtained by blocking the production of Wnt and its secretion and therefore blocks both the canonical and non-canonical pathways. It was revealed that the endogenous Wnt activity must be also blocked to enhance the transcription of cardiac factors [29]. Our results show that the choice of Wnt inhibitor significantly influences the characteristics of the generated hiPSC-CM even in the same architectural environment (i.e., monolayer seeding of cells). We notably found evidence for that cardiomyocytes generated with the 2D-IWR1 did not achieve an optimal sarcomere and electrophysiological maturation as compared to the other protocols. Reciprocally, our results suggest that the use of IWP2 instead of IWR1 result in a better sarcomere maturation as assessed by a lower ssTni and a higher cTni expression, thus suggesting an involvement of the non-canonical Wnt pathways in this process. The molecules from the IWP family block PORCN [23]. PORCN is a membrane-bound O-acyltransferase and it catalyzes the palmitoylation of the serine corresponding to Ser-209 of WNT3A [30]. It is unclear why IWP2 drives a better sarcomere maturation but it could be related to differences in downstream targets of PORCN or to an unknown influence of Wnt secretion. It was recently reported that the Wnt canonical pathway is critical in the early stage of cardiogenesis through mesoderm induction but that the non-canonical Wnt pathways have a greater role in late stages of cardiomyocytes differentiation [31]. The non-canonical Wnt pathway involves the Wnt/Calcium signal transduction cascade that regulates the activities of Calcineurin/Nfat and of CaMKII, two critical pathways for cardiomyocyte growth and regulation of sarcomere functionality. Our results highlight the involvement of Wnt non-canonical pathways in functional cardiomyocytes differentiation and suggest the need to achieve transitory blocking of the pathway for a better sarcomere formation in iPSC-derived cardiomyocytes.

There are five reports of iPSC models of sarcomeric cardiomyopathies [11,12,13,14,15,16]. In most of these reports the cardiomyocytes were derived using the 3D method. We have observed in our study that this method did not achieve the highest level of sarcomere formation and maturation. In another study, published in 2015 [11], titin mutations were studied using 2D protocol and also a microtissue platform (CMT). The use of this microtissue platform can help to study the contractility of the cells and their organization [32]. New methods of tissue engineering were reported in the past years [33,34]. Different matrix scaffolds were used to obtain the most representative and reproductive tissue. Engineered heart tissues (EHT) are a powerful tool to model cardiovascular diseases and can strongly influence the characteristics of the generated cardiomyocytes as measured in our study. However, the constitution of EHT also relies on the method of cardiomyocytes differentiation that is used before reseeding the cells in the tissue. Consequently, our results might also be of strong importance to improve the quality and throughput of tissue engineering.

It is generally viewed that hiPSC-CMs are immature as they do not display the requisite sub-cellular, cellular and tissue-level adult myocytes morphology and the sarcomeric protein content and organization. In this study, we have observed that the use of IWP2 provides cardiomyocytes with a more mature sarcomere, albeit still largely different from the one observed in adult isolated cardiomyocytes from human hearts. In contrast, the expression of main cardiac ion channels was closer to the one observed in adult isolated cardiomyocytes from human hearts. Our results however highlight the lack of current differentiation protocols to achieve this goal. 

We also observed significant electrophysiological differences between protocols with slightly lower resting membrane potential in hiPSC-CM obtained with the 2D-IWR1 protocol and higher action potential duration in both monolayer-based protocols. Previous studies have reported that multiple voltage-gated ion channels are similarly present in hiPSC-CM and adult cardiomyocytes but with significant differences such as reduced inward rectifier potassium currents and the presence of prominent pacemaker currents that support the spontaneous automaticity observed in hiPSC-CM. Our results suggest that differentiation protocol can also significantly influence the AP properties and the function of main cardiac currents as exemplified by the difference in sodium currents in hiPSC-CM obtained with the 2D-IWP2 protocol. Further studies will now be needed to characterize in detail the electrophysiological properties of hiPSC-CM at the single-cell level. In addition, we did not measure the occurrence of abnormal electrophysiological events (such as early after-depolarization events) which could also be influenced by the differentiation protocols.

Finally, to allow for an unbiased evaluation of cell characteristics between protocols, we also designed a new method of high throughput cell imaging for iPSC-derived cardiomyocytes. This tool represents an ideal platform to study and compare a large number of cardiomyocytes in a single acquisition step. We based our method on typical cardiomyocyte staining to simply identify these cells. Using this method, we were especially able to efficiently measure cell sizes in a large number of cells and estimate cell size distribution between the protocols, thus better characterizing cell heterogeneity in response to a given protocol. In addition, other parameters including the number of nuclei and cell morphology can be acquired at the same time. These two parameters have been proposed as maturation markers of iPSC-derived cardiomyocytes. Adult human cardiomyocytes typically present as rod and elongated cells whereas immature cardiomyocytes are more rounded. Similarly, around 25% of adult cardiomyocytes are multinucleated while immature cardiomyocytes are mononucleated. With this approach, we delineate the monolayer-based protocols as providing more mature cells as compared to the aggregation-based protocol. It is, however, important to note that purification methods have recently been proposed to allow enrichment of cardiomyocytes. These methods are particularly suitable with low-yield differentiation protocols such as the 3D protocol. 

## 4. Materials and Methods

### 4.1. Human iPSC Derivation

A total of four different iPSC clones reprogrammed by different methods from skin fibroblasts originating from unrelated individuals were used (Figure S1). Firstly, we used a hiPSC line (named 31.3) derived from the skin biopsy of a volunteer as previously described [19] and provided by the Cardiovascular Research Center at Mount Sinai, NY, USA. Then, we generated three different new clones coming from two other donors. Human dermal fibroblasts (HDFa) were purchased from Life Technologies (# C-013-5C lot 1168064, Carlsbad, CA, USA) and Cell Application inc (# 106-05a lot 1392, San Diego, CA, USA) and were used to generate three other clones (clones 3.7 and 3.8 originating from HDFa purchased at Life Technologies and one other clone named 4.9 originating from HDFa purchased at Cell Application).

The fibroblasts were cultured with fibroblast growth medium containing DMEM medium (Life Technologies, Carlsbad, CA, USA), 10% of FBS (Sigma Aldrich, Saint-Louis, MO, USA), 10 ng/mL FGF-2 (R&DSystems, Minneapolis, MN, USA) and 1% penicillin-streptomycin (Life Technologies, Carlsbad, CA, USA). Cell media were changed every 2 days. Reprogramming of fibroblasts into iPSCs was carried out using episomal vector. The HDFa were trypsined and counted, 6 × 10^5^ cells were electroporated with Nucleofector II (Lonza, Bale, Swiss) with 100 µL of NHDF Nucleofector^®^ Kit (Lonza) and 1 µg/µL of each episomal vector (ref : 27077, 27078, 27080, Addgene, Cambridge, MA, USA). The cells were seeded (100,000 cells/well) in 6-well plates coated with matrigel matrix (Corning, New York, NY, USA) and cultured with fibroblast growth medium for 5 days. On Day 6 the medium was switched to reprogramming medium containing Essential 6 medium (Life Technologies) and 10 ng/mL FGF2. The media was changed every 2 days. Around 25–30 days post-electroporation the iPS colonies appeared. The iPSCs colonies were picked and expanded in mTeSR1 (Stemcell technologies, Vancouver, BC, Canada) on matrigel matrix coated plates. The iPSCs were passed once a week manually using a Lynx Microscope (Vision Engineering, New Milford, CT, USA) and the culture medium was changed daily. The cells were cultivated in 5% CO_2_, 5% O_2_ at 37 °C. The expression of pluripotency genes was verified by qPCR, immunostaining and alkaline phosphatase staining. In addition, a predictive qPCR analysis using TaqMan^®^ hPSC Scorecard™ Assay was performed to assess the ability of each iPSC clone to differentiate into the three germ line lineages. The karyotype of the iPSC control cell line was normal. 

### 4.2. Aggregation Cardiac Differentiation—3D Protocol

iPSCs were passed and cultured 7 days before differentiation then incubated with PBS for 5 min, detached with a cell scraper and induced into mesoderm in suspension on low-attachment plates (# 056263, Nunc, Roskilde, Denmark) in mTeSR1 supplemented with BMP4 (10 ng/mL, R&D Systems, Minneapolis, MN, USA) and blebbistatin (5µM, Sigma-Aldrich, Saint-Louis, MO, USA). After 24 h, the medium was changed to 3D basal differentiation medium composed by StemPro34 SFM (Life Technologies), ascorbic acid 50 µg/mL (Sigma-Aldrich) and 2 mM GlutaMax-I (Life Technologies) supplemented with BMP4 (10 ng/mL) and Activin A (25 ng/mL, R&D Systems) for 48 h and then switched to 3D basal differentiation medium for another 36 h. To induce the cells into cardiac progenitors, the small molecule IWR1 (Sigma-Aldrich) was added to the media on day 4.5. The differentiated cells were maintained in 3D basal differentiation medium from day 8 until day 25. The cells were cultivated in 5% CO_2_ at 37 °C. 

### 4.3. Monolayer Cardiac Differentiation—2D IWR1 and 2D IWP2

iPSCs were passed and cultured 7 days before differentiation with StemPro EZP disposable stem cell passaging tool (Life Technologies) to obtain clumps and then they were plated on a 12-well plate. The monolayer differentiation is initiated when the hiPSC obtain 70–80% of confluence. The cells were cultured in 2D differentiation medium composed of RPMI1640 medium (Life Technologies) and B27 supplement minus insulin (Life Technologies). The mesoderm progenitors were induced by supplementing the 2D differentiation medium with 6 µM CHIR99021 (Abcam, Cambridge, UK) for 3 days. The cardiac progenitors were induced on day 3 with 2D differentiation medium supplemented with either a Wnt inhibitor IWR1 (Inhibitor of Wnt Response, 2.5 µM) or IWP2 (Inhibitor of Wnt Production, 5 µM) (Tocris, Bristol, USA) for 2 days. The differentiated cells were maintained in 2D differentiation medium from day 5 until day 25. The cells were cultivated in 5% CO_2_ at 37 °C.

### 4.4. Flow Cytometry

On day 27 ± 2 of the differentiation the cells were detached and dissociated with Detachment Kit 2 (Promocell, Heidelberg, Germany) centrifuged 5 min at 200 g then fixed with paraformaldehyde (4%) for 10 min at RT (Sigma-Aldrich) and permeabilized with 90% cold methanol for 15 min at +4 °C . The cells were washed 3 times with PBS and then stained with Anti-Cardiac Troponin T-APC 1:100, recombinant human IgG1, clone REA400 (MiltenyiBiotec, Bergisch Gladbach, Germany) or CTL-I APC 1:100, Monoclonal REA Control (I) antibody human, clone REA293 (MiltenyiBiotec) diluted in PBS plus 0.1% Triton X-100 and 0.5% BSA for 45min at RT in the dark. The cells were washed and resuspended with PBS plus 0.5% BSA and collected on MACSQuant^®^ Analyzer 10 (MiltenyiBiotec) and analyzed using FlowJo.

### 4.5. Automated Cell Imaging

On day 27 ± 2 of differentiation the cells were detached and dissociated with a Detachment Kit 2 and cultured for 7 days on matrigel-coated plates. The cells were fixed in paraformaldehyde (4%) and permeabilized in blocking/permeabilization buffer (2% BSA, 0.5% Triton-X-100 in PBS) for 45 min then incubated overnight at +4 °C with rabbit polyclonal anti-Cardiac Troponin T antibody (Ref #ab45932 Abcam) diluted at 1:500 in blocking/permeabilization buffer. The cells were washed 3 time in PBS and incubated with 488-Alexa-conjugated secondary antibody (Life Technologies) and DAPI (SantaCruz, Dallas, TX, USA) both diluted at 1:1000 in blocking/permeabilization buffer for 45 min at RT. The plates were scanned and analyzed using the Cell Insight CX5 Platform (ThermoFisher Scientific). This automated cell imaging strategy was used to evaluate the morphology of the cells, their surface size as well as the number of nuclei per cell. To define the cell geometric morphology, a specific algorithm was defined using the CX5 cell imaging program (HCS studio) and based on the aspect ratios between two axes traced in the cells. A ratio below 1.5 indicated a rounded cell (called round) whereas ratios >1.5 indicated elongated cells (called long).

### 4.6. Immunocytochemistry

iPSC were cultured on 4 Well Culture Slide (Corning) for 3 days and then fixed in paraformaldehyde (4%) and permeabilized in blocking/permeabilization buffer (2% BSA, 0.5% Triton-X-100 in PBS) for 45 min then incubated overnight at +4 °C with primary antibodies diluted in blocking/permeabilization buffer. The cells were washed 3 times in PBS and incubated with Alexa-conjugated secondary antibodies and DAPI both diluted at 1:1000 in blocking/permeabilization buffer for 45 min at RT. The images were acquired using a Epifluorescence Microscope (Eclipse TE300, Nikon, Amsterdam, the Netherlands). The following antibodies were used: rabbit anti-Nanog (#4903S, 1:200, Cell Signaling-Ozyme, Beverly, MA, USA), rabbit-Oct4 (#3576-100, 1:200, Biovision, Cliniscience, Mountain View, CA, USA), rabbit anti-Sox2 (#AB5603, 1:200, Millipore, Ballerica, MA, USA), mouse anti- Tra-1-60 (#MAB4360, 1:100, Millipore), mouse anti- Tra-1-81 (#MAB4381, 1:100, Millipore), mouse Anti-SSEA4 (#sc-21704, 1:100, Santa Cruz, Dallas, TX, USA).

On day 27 ± 2 of differentiation the cells were detached and dissociated with a Detachment Kit 2 and cultured for 7 days on matrigel-coated coverslips. The cells were fixed in paraformaldehyde and permeabilized in blocking/permeabilization buffer (2% BSA, 0.5% Triton-X-100 in PBS) for 45 min then incubated overnight at +4 °C with mouse monoclonal anti-α-Actinin (Sarcomeric) antibody clone EA-53 (#A7732, Sigma-Aldrich) diluted at 1:1000 in blocking/permeabilization buffer. The cells were washed 3 times in PBS and incubated with 546- Alexa-conjugated secondary antibody (# A10036, Life Technologies) and DAPI (Santa Cruz) both diluted at 1:1000 in blocking/permeabilization buffer for 45 min at RT. The images were acquired using DeltaVision Elite Deconvolution System (GE Healthcare, Chicago, IL, USA) and analyzed using Fiji Software.

### 4.7. Western Blot Analysis

Cells and control cardiac and muscular tissues were lysed in RIPA buffer (150 mM NaCl, 0.1% Triton X-100, 0.5% sodium deoxycholate, 0.1% SDS, 50 mM Tris-HCl, pH 8.0, Protease inhibitors). Proteins were separated on 12% NuPAGE™ Novex™ 12% Bis-Tris Protein Gels (Life Technologies) on denaturing conditions and transferred to a nitrocellulose membrane by iBlot System (Life Technologies). After blocking with 5% milk in PBS-Tween 0.1%, the membrane was incubated with primary antibodies overnight at 4 °C. The following antibodies were used: mouse monoclonal anti-TNNI1 antibody clone 12F10 (#ab8293, dilution 1:1000, Abcam), rabbit polyclonal anti-Cardiac Troponin I (#ab47003, dilution 1:500, Abcam), rabbit anti-GAPDH (#ab9485, dilution 1:2000, Abcam). The membrane then was then washed, incubated with an anti-mouse/rabbit peroxidase-conjugated secondary antibody (1:1000, Cell Signaling) at room temperature for 1 h, and developed on ImageQuant LAS 4000 (GE Healthcare, Chicago, IL, USA). The quantification was performed using Fiji Software and normalized to GAPDH.

Heart tissue was obtained from a patient undergoing surgery for left ventricular assistance device implantation and skeletal muscle tissue from a patient referred for a muscle biopsy. The tissues were immediately frozen. The program was approved by the ethics committee (CPP Ile de France 1, ID 2014-sept-13691).

### 4.8 Quantitative RT-PCR

Relative gene expression was determined using a two-steps quantitative real-time PCR method. Total RNA was isolated on day 30 of differentiation with the PureLink^®^ RNA Mini Kit (Life Technologies) and reverse-transcribed using the Maxima First Strand cDNA Synthesis Kit for RT-qPCR (Life Technologies). Quantitative RT-PCR was performed with SYBR Select Master Mix (Life Technologies) on the LightCycler^®^ 480 Instrument II (Roche Life Science, Bale, Swiss). Fold changes in gene expression were determined using the comparative CT method (dd*C*_t_) with normalization to the housekeeping gene RPL32. All the primers used are listed in Table S1.

### 4.9. Patch Clamping

Dissociated iPSC derived CMs differentiated during 25 days were directly seeded at the density of 7 × 10^5^ cells on glass coverslips (#354086, Corning) coated with fibronectin and then incubated for 3 to 8 days in cultivation medium in a 5% CO_2_ incubator at 37 °C.

Patch-clamp experiments were performed at 37 °C and the cardiomyocytes were continuously perfused with an external solution containing (in mM): 140 NaCl, 5 KCl, 1 CaCl_2_, 1 MgCl_2_, 10 glucose, 10 HEPES. pH was set to 7.4 with NaOH 1M. The patch pipettes were pulled from thick-walled borosilicate glass capillaries (Harvard Apparatus, Edenbridge, UK) on a DMZ-universal electrode puller (Zeitz-Instruments GmbH). Electrode impedance was 4–6 MOhms when filled with an internal solution containing (in mM): 110 Kaspartate, 20 KCl, 1 MgCl_2_, 0.1 Na-GTP, 5 Mg-ATP, 5 phosphocreatine, 1 EGTA, 10 HEPES. pH was adjusted to 7.2 with KOH 1 M. Data were all corrected for liquid junction potentiel (15.4 mV).

Ionic currents and action potentials were recorded on isolated spontaneous beating CMs in the whole-cell configuration of the patch-clamp technique using digidata 1550/Multiclamp 700B (Molecular Devices, Sunnyvale, CA, USA) for data amplification and acquisition. Data were acquired at 10 KHz and low-pass filtered at 5 kHz using Clampex software (pClamp 10.5, Molecular Devices).

The same following protocol was applied to all CMs (*n* = 427): upon seal formation and following patch membrane break, a 50 ms test pulse from a holding potential of −80 to 0 mV was applied in voltage–clamp mode in order to record Na^+^-current maximal amplitude. The patch was then switched to current–clamp mode and spontaneous action potentials (APs) were recorded for 1min at resting potential without injecting any current.

For currents and AP analysis, Clampfit software was used (pClamp 10.5, Molecular Devices). Na^+^-current density was obtained by normalizing Na^+^-current maximal amplitude to the cell capacitance and expressed in pA/pF. For AP analysis, common electrophysiological characteristics such as action potential duration at 50% of repolarization (APD_50_), action potential amplitude and resting membrane potential (RMP) were calculated.

### 4.10. Statistical Analysis

All analyses were performed using Prism 6.0 (GraphPad, San Diego, CA, USA). Continuous data are presented as Mean ± SEM. Continuous variables were compared using 1-way analysis of variance followed by a Tukey’s multiple comparison test. Exact tests were used for experiments with *n* < 5. Chi square testing was used for frequency comparisons. A *p*-value ≤ 0.05 is considered significant. For comparison of mRNA expression, the values for the human LV tissue were reported as standard but were excluded from the statistical analysis where results obtained from the 3 different protocols were statistically compared. 

## 5. Conclusions

In conclusion, by directly comparing methods for cardiomyocytes generation from different iPSC lines and using high-screening tools, we found that differentiation of cardiac myocytes with 2D monolayer-based protocols and the use of the Wnt inhibitor IWP2 allows the production of higher yield of cardiac myocytes that have more suitable characteristics to study sarcomeric cardiomyopathies. 

## Figures and Tables

**Table 1:** Main electrophysiological parameters. (*) indicates significances levels of *p* < 0.05 and (**) *p* < 0.01 3D vs. 2D-IWR1. (^$^) indicates significances levels of *p* < 0.05 and (^$$^) *p* < 0.01 3D vs. 2D-IWP2 (^£^) indicates significances levels of *p* < 0.05 and (^££^) *p* < 0.01 2D-IWR1 vs. 2D-IWP2.

Protocol	Number of Cells (n)	RMP (mV)	Frequency (Hz)	APA (pA)	APD50 (ms)	APD90 (ms)	Number of Cells (n)	INa (pA/pF)
3D	48	−75.54 ± 8.88	0.63 ± 0.13	98.29 ± 16.8	490.46 ± 428.46	536.75 ± 450.9	47	−67.85 ± 53.9
2D-IWR1	86	−68.55 ± 8.14 **	0.44 ± 0.06	94.5 ± 13.4	540.53 ± 357.89	633.91 ± 397.55 *	75	−65.76 ± 60.06
2D-IWP2	70	−69.66 ± 10.67	0.40 ± 0.04	96.26 ± 15.8	591.55 ± 399.25	719.61 ± 496.02 ^$$^	57	−83.79 ± 48.96 ^$,££^

## Supplementary Materials

Supplementary materials can be found at www.mdpi.com/1422-0067/18/6/1173/s1.

## Abbreviations

APA	Action potential amplitude
APD	Action potential duration
EB	Embryoid body
cTNT	Cardiac troponin T
hiPSC	Human induced pluripotent stem cells
hiPSC-CM	Human induced pluripotent stem cells derived cardiomyocytes
RMP	Resting membrane potential
