# Supplementary material for: Differential Sarcomere and Electrophysiological Maturation of Human iPSC-Derived Cardiac Myocytes in Monolayer vs. Aggregation-Based Differentiation Protocols
Source: Int J Mol Sci. 2017 Jun 1;18(6):1173. doi: 10.3390/ijms18061173 (PMC5485997; doi:10.3390/ijms18061173)
Supplement: Supplementary file 1 [file ijms-18-01173-s001.pdf]

SUPPLEMENTARY FIGURES

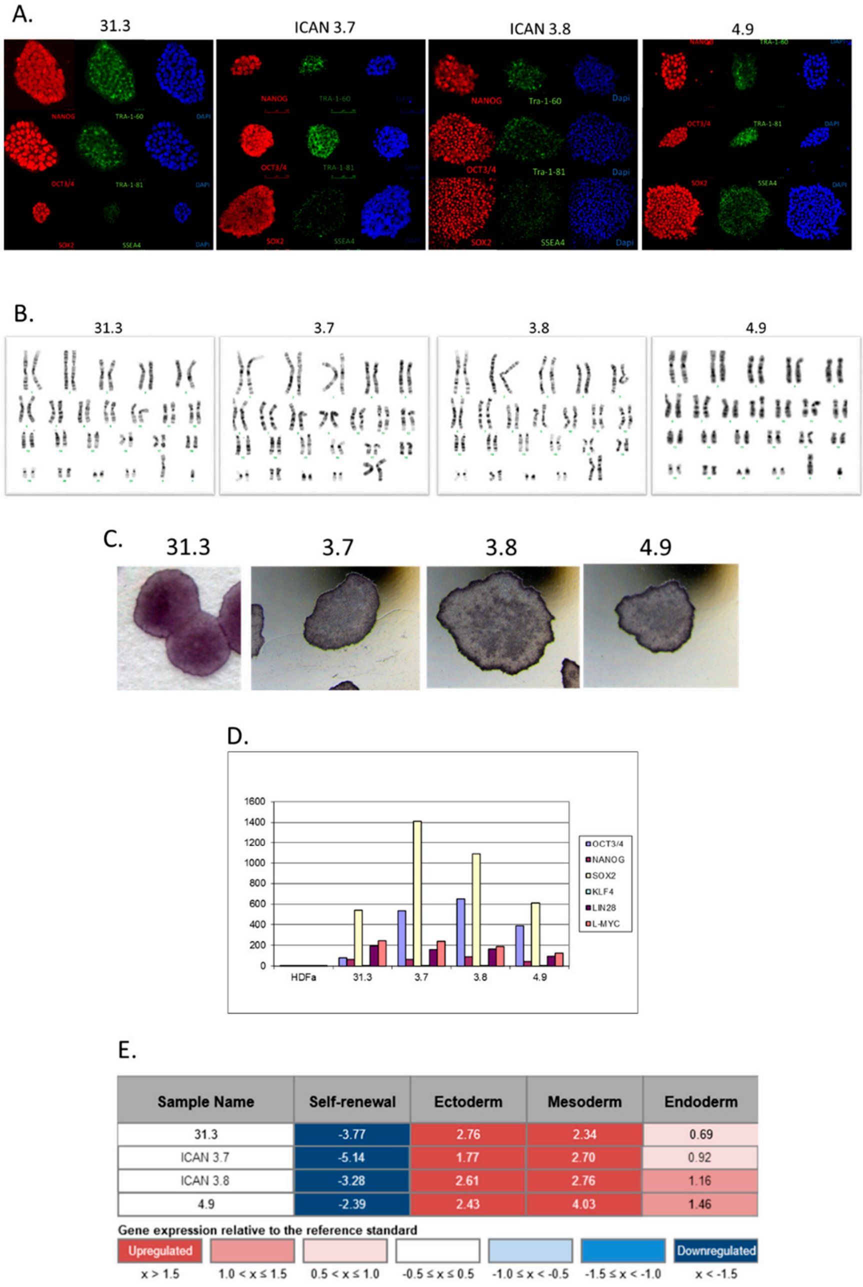

Figure S1: Pluripotency of iPS control cell lines A. Immunostaining of iPS with antibodies directed against the pluripotency markers NANOG, OCT4, SOX2 (red), TRA1-60 TRA1-81 and SSEA3/4 (green). Nuclei were stained with DAPI (blue). **B**. Normal karyotyping of cell lines **C.** Positive alkaline phosphatase staining **D.** RT-PCR showing the expression of pluripotency genes compared human dermal fibroblasts and control iPS cell line. **E.** Scorecards™ analysis assessing pluripotency and trilineage differentiation.

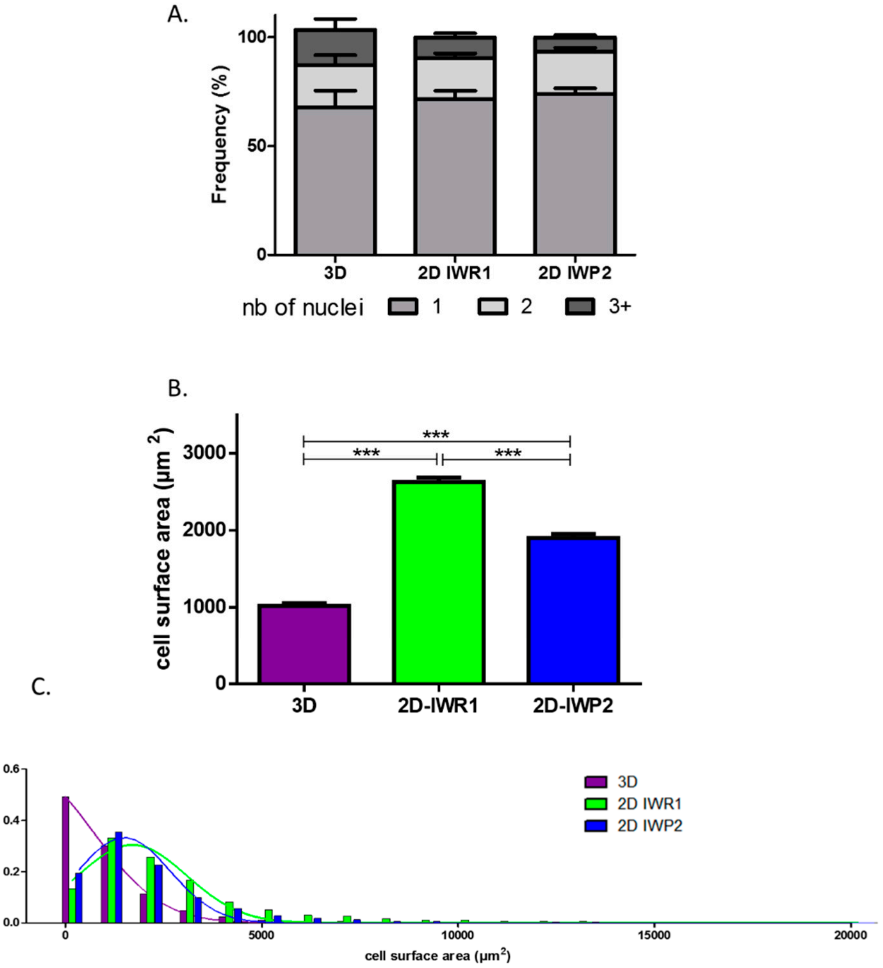

Figure S2 related to Figure 2: Automated cellular imaging to analyze the cell size and nucleation of the round cells population. A. Frequency of number of nuclei in the round cells for each protocol **B.** Cell size comparison in the round population **C.** Cell size distribution in the three protocols in the round population.

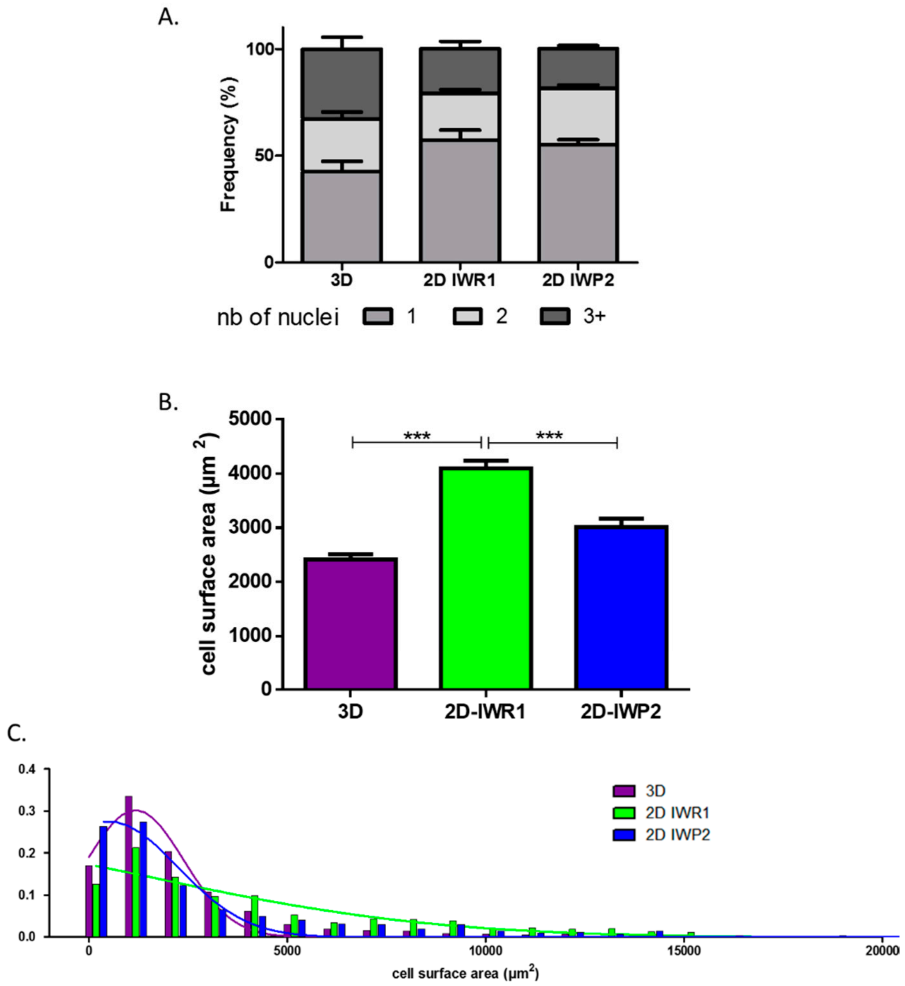

Figure S3 related to Figure 2: Automated cellular imaging to analyze the cell size and nucleation of the long cells population. A. Frequency of number of nuclei in the long cells for each protocol **B.** Cell size comparison in the long cell population **C.** Cell size distribution in the three protocols in the long population.

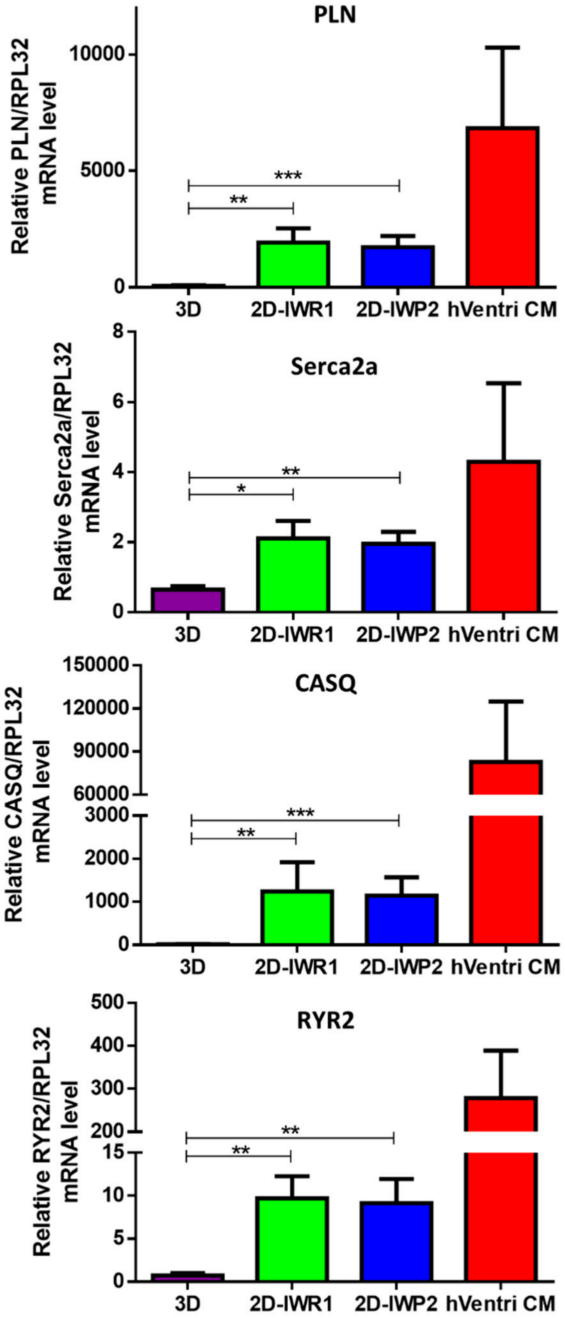

Figure S4: mRNA expression of calcium cycling proteins. Quantitative PCR results for the mRNA expression of *PLN, CASQ, Serca2a,* and *RYR2.* Adult LV tissue was used as a positive control. n=8 to 12 differentiations, *p<0.05 , **p<0.01, ***p<0.001

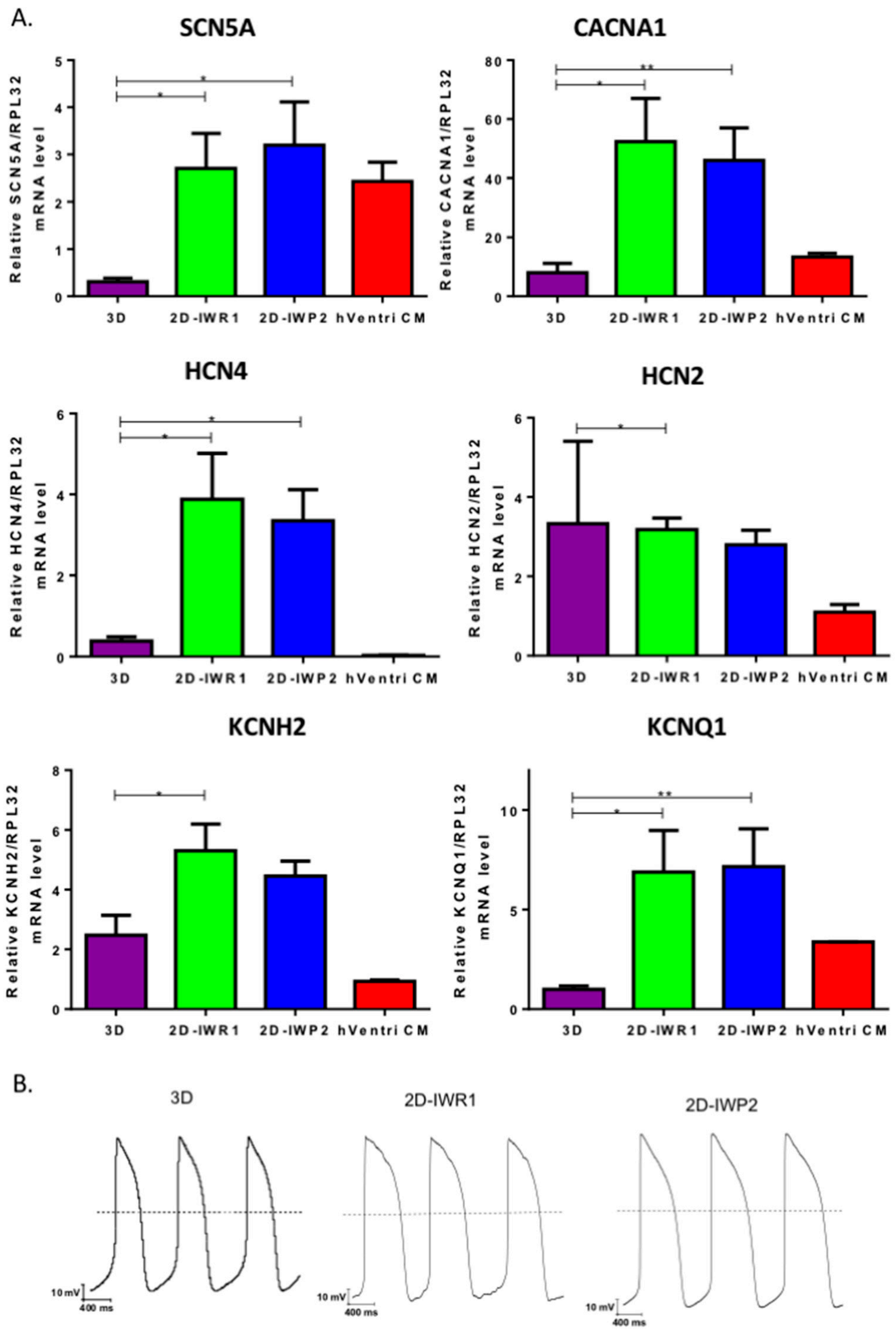

Figure S5: Action potentials and expression of cardiac ion channels. A. Expression of main human cardiac ion channels**.** Quantitative PCR results for the expression of *SCN5A, CACNA1, HCN2, HCN4, KCNQ1,* and *KCNH2.* Adult LV tissue is used as a positive control. n=8 to 12 differentiations, *p<0.05, **p<0.01 **B.** Typical action potentials recorded in hiPSC-CM according to differentiation protocols

SUPPLEMENTARY TABLES

Table S1: Sequences of primers

NAME	FOWARD SEQUENCE (5' > 3')	REVERSE SEQUENCE (5' > 3')
α-MHC	TCTCCGACAACGCCTATCAGTAC	GTCACCTATGGCTGCAATGCT
β-MHC	GGCAAGACAGTGACCGTGAAG	CGTAGCGATCCTTGAGGTTGTA
cTNi	CCAACTACCGCGCTTATGC	CTCGCTCCAGCTCTTGCTTT
ssTNi	AACCCAAGATCACTGCCTCCCG	CGCTTGAACTTCCCACGGAGGT
PLN	CTCACTCGCTCAGCTATAAGAAG	AGAGAAGCATCACGATGATACAG
SERCA2A	ACCCACATTCGAGTTGGAAG	CAGTGGGTTGTCATGAGTGG
CASQ2	GAGCTTGTGGCCCAGGTCCT	GATCTCCACTGGGTCTTCAA
RYR2	GGTGGTCATCGAACACTCCT	CGAGCAATACAACCTGACCA
KCNH2	CCCAACACCAACTCAGAGAAGATC	TAGCATACATGAGGGAGCCAATG
KCNQ1	GCCTCCTGCTTCTCTGTCTTT	CTTCTGCCTCTGCTTCTGCT
CACNA1	CGAAGTAGGTGGAGTTGACCAC	GCAGGAGTACAAGAACTGTGAGC
HCN2	ATCCAGTCGCTGGACTCCT	GATCTTCTGGCGGAAGTCAG
HCN4	TCTACTCGCTGAGCGTGGACAA	GAGTTGAGGTCGTGCTGGACTT
